# Unmasking Greenwashing: Mapping Hijacked Medicine Journals to the Sustainable Development Goals

**DOI:** 10.34172/apb.43763

**Published:** 2024-09-24

**Authors:** Mihály Hegedűs, Mehdi Dadkhah, Lóránt Dénes Dávid

**Affiliations:** ^1^Tomori Pál College, Chamber of Hungarian Auditors, Budapest, Hungary.; ^2^Amrita School of Engineering, Amrita Vishwa Vidyapeetham, Amritapuri, Kerala, India.; ^3^John von Neumann University, Faculty of Economics and Business, Department of Tourism and Hospitality, HU-6000 Kecskemét, Hungary.; ^4^Hungarian University of Agriculture and Life Sciences (MATE), Institute of Rural Development and Sustainable Economy, Department of Sustainable Tourism, HU-2100 Gödöllő, Hungary.; ^5^Eötvös Loránd University, Faculty of Social Sciences, Savaria University Centre, Savaria Department of Business Economics, HU-9700 Szombathely, Hungary.; ^6^Széchenyi István University, HU-9026 Győr, Hungary.

**Keywords:** Hijacked journals, Medicine, Predatory journals, Publication ethics, Scientific ranking, Sustainable development goals, Circular economy

## Abstract

**Purpose:**

Hijacked journals are journals managed by cybercriminals that mimic the original journal and publish manuscripts without peer review, charging a fee to the author. Although there is literature on hijacked journals, there is a gap in the content of published papers in the hijacked journals. This study aims to analyze the content of published papers in hijacked journals to assess their alignment with various Sustainable Development Goals (SDGs).

**Methods:**

About 21 medicine journals have been investigated and about 3300 published manuscripts in them analyzed in terms of SDGs using the text-based analyzing method.

**Results:**

The findings indicated that published manuscripts fit in the categories of SDG 01, SDG 03, SDG 11, and SDG 16 where SDG-03 is most dominant.

**Conclusion:**

The awareness about the problem of hijacked journals is critical, especially for developing countries, to eliminate the negative effects of these journals. It is the first research that discusses the negative effect of hijacked journals by considering SDGs and sheds light on the phenomenon.

## Introduction

 Reputable academic journals, for example, journals indexed in Web of Science^TM^ (WoS) Journal Citation Report and Scopus, are faced with a high volume of submissions and limited space for publishing papers. As a result, they only accept the most qualified papers and reject many submitted manuscripts.^1^ Cyber fraudsters, referred to in this paper as hijackers, found this situation an opportunity to launch new types of fraud by developing fake websites for indexed journals. Typically, hijackers search for indexed journals that do not have a website (print-only journals), or their websites have a low ranking in the search engines, so it is difficult to find the journal’s website. Hijackers then develop a website for the target journal with good search engine optimization (SEO) and claim that this hijacked website is the original website of the indexed journal. When researchers search for the name of the reputable, indexed journal in various search engines, they find the fake website. This paper refers to these as hijacked journals. In other words, hijacked journals are the second fake website(s) for original authenticated journals that have been created and managed by hackers.^2^ This process has been called journal hijacking.^3,4^

 Researchers searching for reputable journals may search the ISSN in WoS to ensure the journal is indexed. However, this will not be enough to deal with hijacked journals because they use the exact description of the original journal, including its title, ISSN, and other information about the journal. If a bibliographic database such as WoS provides a link to the original website of the journal, the hijacking attempt will likely fail. In 2015, however, Dadkhah detected the URLs of some hijacked journals in the WoS database with the journal description. Thus, for these journals, researchers searching for a journal would be directed to the hijacked version.^5^ Dadkhah proposed a method for detecting this new kind of advanced journal hijacking. Dadkhah found that hijackers monitor journal websites, and when a domain of a journal website has expired, they register the domain and gain control of that ceased journal ^5^. Other names for this phenomenon include journal phishing, and cloned or robbed journals.^6^

 Some researchers confuse predatory journals with hijacked journals and think the two are the same, but they are not. A predatory journal is a journal that does not adhere to editorial and publishing standards. Predatory journals have their name and ISSN, even though they may mimic the names of some reputable and legitimate journals.^7,8^ Hijacked journals, in contrast, mimic a journal with its title and ISSN to deceive researchers. These journals publish any manuscript, without peer review, and charge authors a fee to publish article.^1^

## Sustainable Development Goals

 The SDGs are a set of global goals adopted by the UN in 2015, as part of the 2030 Agenda for Sustainable Development. The SDGs are a framework for global action to end poverty, protect the planet, and ensure that all people have the opportunity to live peaceful and prosperous lives.^9,10^ There are 17 SDGs in total, all interconnected and interdependent. The SDGs are no poverty (SDG 1), zero hunger (SDG 2), good health and well-being (SDG 3), quality education (SDG 4), gender equality (SDG 5), clean water and sanitation (SDG 6), affordable and clean energy (SDG 7), decent work and economic growth (SDG 8), industry, innovation and infrastructure (SDG 9), reduced inequalities (SDG 10), sustainable cities and communities (SDG 11), responsible consumption and production (SDG 12), climate action (SDG 13), life below water (SDG 14), life on land (SDG 15), peace, justice, and strong institutions (SDG 16), and partnerships for the goals (SDG 17).^9^

 The importance of the SDGs lies in their potential to address some of the most pressing challenges facing the world today. These challenges include poverty, inequality, environmental degradation, and the impacts of climate change, among others. The SDGs aim to create a more sustainable and equitable world for present and future generations by addressing these challenges. The SDGs are important for everyone, affecting all aspects of our lives and the world. Governments, businesses, civil society organizations, and individuals all have a role to play in achieving the SDGs. The SDGs also provide a common framework for countries to measure progress and hold themselves accountable for meeting their commitments. Various research groups analyze the progress of SDGs-related research in different fields based on regions.^11-15^

## Hijacked journals and SDGs

 Hijacked journals can hurt research that supports SDGs by publishing manuscripts that are related to the United Nations (UN) SDGs. Open access (OA) publishing has an impact on SDGs,^16^ and hijacked journals mainly mimic the OA publishing model to publish papers but without the required peer review and publishing process. Open access publishing provides access to cutting-edge knowledge (SDG 9). New research findings disseminated in OA papers can support clean energy, sustainable agriculture, and predict risks (SDG 2, 7, and 11); improve the equality of researchers across countries to science (SDG 6, and 10); provide access to transparent and accountable data (SDG 16); and can move forward all SDGs and their policy-making.^16^ Hijacked journals mainly mimic this OA model, and publish papers that may contribute to SDGs. These published papers are not peer reviewed and can threaten the integrity of science and disseminate error and misleading information when receiving citations from the side of research.

 Abalkina found some indexed content from hijacked journals in bibliographic databases that have been indexed by mistake or penetration of bibliographic databases.^17^ However, this content was removed when the hijacked nature of the journal was confirmed.^17^ Hijacked journals claim that they are indexed in bibliographic databases but they are not indexed. The original journal is indexed and only content from the original journal. The hijacked version only mimics the original one. When researchers publish in hijacked journals (intentionally or by being deceived), the number of indexed papers for that country will decrease in these bibliographic databases. As a result, the scientific rank of that country and its universities will be negatively impacted.^18^ There are some ranking systems to credit universities and countries based on SDGs-related research,^19^ so hijacked journals will negatively impact the SDGs ranking of universities.

 The published papers in hijacked journals will not be indexed (or will be removed upon detection), and most hijackers close their websites with the hijacked journals after some time,^20^ thus losing these potential scientific contributions to SDGs. Hijacked journals are a risk to the integrity of science, as they publish non-peer-reviewed content.^1^ Others may cite this information as the articles appear to be peer-reviewed academic papers. This can lead to the propagation of pseudo-science and errors from published SDGs-related papers in hijacked journals to other research that supports SDGs in legitimate journals.

 There is no study to examine how much the published papers in hijacked journals are related to SDGs. Indeed, most of the published papers on the topic of hijacked journals introduce the problem of these journals and try to provide information for the detection of them. Current research aims to examine the content of published papers in hijacked journals to assess their alignment with various SDGs.

## Literature review

 The search in bibliographic databases (both WoS and Scopus) did not return any publications regarding hijacked journals and sustainable development (as of March 2024). This is a gap in this regard, and it reveals the importance of discussing hijacked journals concerning SDGs. A search on the term “hijacked journal*” in Scopus by article titles returned 23 documents. A search in Google Scholar revealed some additional publications. Most studies in this area were done by a few researchers, including *Dadkhah, Maliszewski, Borchardt, Abalkina, Jalalian, Davarpanah Jazi, Moussa, etc*. Most publications focus on increasing awareness about the phenomenon with limited discussion on the negative impact of hijacked journals or methods for detecting hijacked journals.

 Although there are limited publications on hijacked journals, the extent of the potential impact is not limited: hijacked journals can negatively affect the scientific rank of more than 71% of countries worldwide.^18^ The analysis of the marketing field indicates that published papers in questionable journals could receive citations from SSCI-indexed marketing journals.^21^ The problem is even more critical when the content of hijacked journals, which are not peer-reviewed, is indexed in reputable bibliographic databases (by mistake or deception by hijackers).^5,22^

## Methodology

 The data for the study are the published papers in the hijacked version of medicine journals as introduced in research by Dadkhah and his colleagues.^23^ These data are based on known hijacked journals as listed in Retraction Watch Hijacked Journal Checker on 15 Jan 2023 16 and alignment of them based on Scimago’s main subject area (https://www.scimagojr.com) on medicine category. Twenty-one hijacked medical journals have been identified, and their published papers have been downloaded as soon as possible without any filter on the date of publication. This period has been selected because most hijacked journals are only available for a short time; once the expected income is received, the hijacker may close the website to prevent detection of their identity or maybe abandon it.

 Collecting these data, the title and abstract of the downloaded articles were extracted to shape the dataset. Different methods exist to identify papers related to each SDGs, such as Elsevier, SIRIS, Aurora, etc.^24^ Elsevier published a report entitled “The power of data to advance SDGs” in 2020. This report discussed SDG-related publications from 2015 through 2019 across various fields.^25^ The Elsevier method identified SDG publications based on Boolean search queries to find SDG-related keywords in the papers. The source of data was Scopus.^24,26^ The SIRIS also checks by using a set of terms related to each SDG by the UN, but it also searches for keywords that semantically may be related to the main SDG terms. It uses a machine learning model based on neural networks to conduct this process.^24,27^ Aurora also uses Boolean search strings based on terms in the UN policy documents and Scopus data, but it is not fully developed for global analysis. The future version of Aurora will include new terms from updated UN documents, synonyms, keywords, combinations, and terms retrieved through survey data.^28,29^

 As there is no best method to identify texts that are related to each SDG,^12,30^ the current study used all three methods, including Elsevier, SIRIS, and Aurora, to map articles in the hijacked journals to the SDGs. These methods help to understand which publications contribute to which SDGs. These methods have been implemented by using the R programming language. The appendix shows the related R libraries used for the methodology section.

## Findings

 The list of used medicine hijacked journals is shown in Table 1.^23^ Some hijacked journals were inactivated, and some did not provide free access to the published papers. Finally, A dataset of 3384 papers from hijacked journals was compiled. Table 1 provides the names of the hijacked versions of the journals, and the number of downloaded articles.

**Table 1:** The list of used medicine hijacked journals^23^

**Hijacked Journal Title**	**URL (Hijacked)**	**Number of downloaded papers**
Acta Biomedica	https://mattiolli1885journals.com	171
Acta Biomedica	https://mattioli1885journal.com	156
Azerbaijan medical journal	https://www.azerbaijanmedicaljournal.com	The website is not available.
Azerbaijan medical journal	https://www.azerbaijanmedicaljournal.life	195
Azerbaijan medical journal	https://www.azerbaijanmedicaljournal.net	198
Bulletin of National Institute of Health Sciences	https://www.healthsciencesbulletin.com	91
Chinese Journal of Medical Genetics	http://zhyxycx.life	The website is not available.
Community Practitioner	https://commprac.com	The website is not available.
International Medical Journal	https://www.seronijihou.com	The full text required a subscription.
Journal of Clinical Otorhinolaryngology, head, and neck surgery	www.lcebyhkzz.cn	886
Journal of Korean Academy of Psychiatric and Mental Health Nursing	https://mhnursing.or.kr/index.php/JKPMHN	92
La Prensa Medica Argentina	https://www.scitechnol.com/laprensamedica.php	The journal is not available.
New Armenian Medical Journal	https://www.newarmenianmedicaljournal.com	The website is not available.
Pakistan Heart Journal	https://pkheartjournal.com	412
Sapporo Medical Journal	https://www.maejournal.com	376
Tagliche Praxis	https://www.taglichepraxis.com	The website is not available.
Teikyo Medical Journal	https://www.teikyomedicaljournal.com	807
Turkish Journal of Physiotherapy and Rehabilitation	https://turkjphysiotherrehabil.org	The website is not available.
Turkish Journal of Physiotherapy and Rehabilitation	https://turkjphysiotherrehabill.org	The full text required a subscription.
Chinese Journal of Otorhinolaryngology Head and neck surgery	https://www.dev1.zhebyhkperiodicalscn.net	The website is not available.
Chinese Journal of Otorhinolaryngology Head and neck surgery	https://www.zhebyhkperiodicalscn.net	The website is not available.

**Figure 1:**
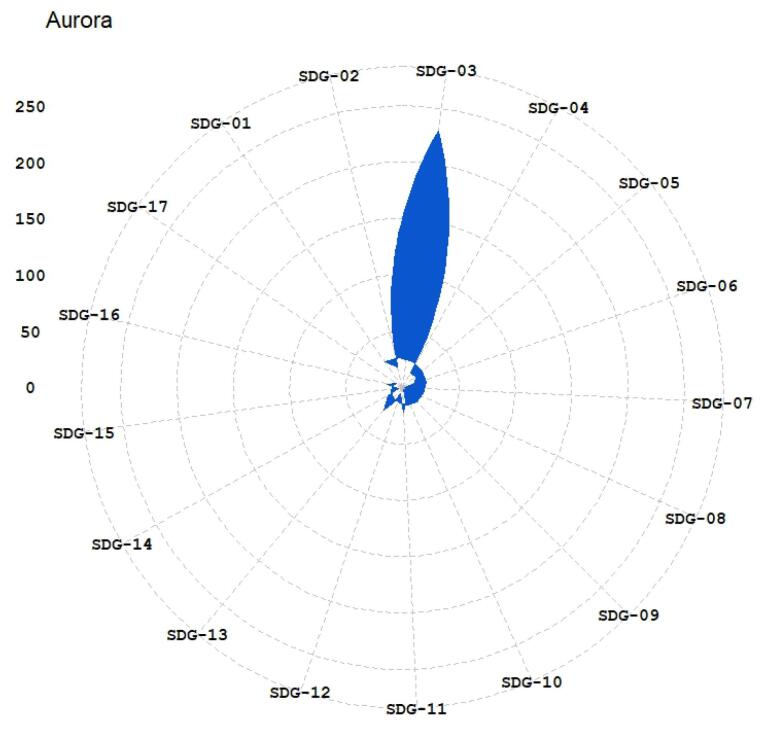
The published papers in hijacked journals and their map to SDGs (using Aurora)

**Figure 2:**
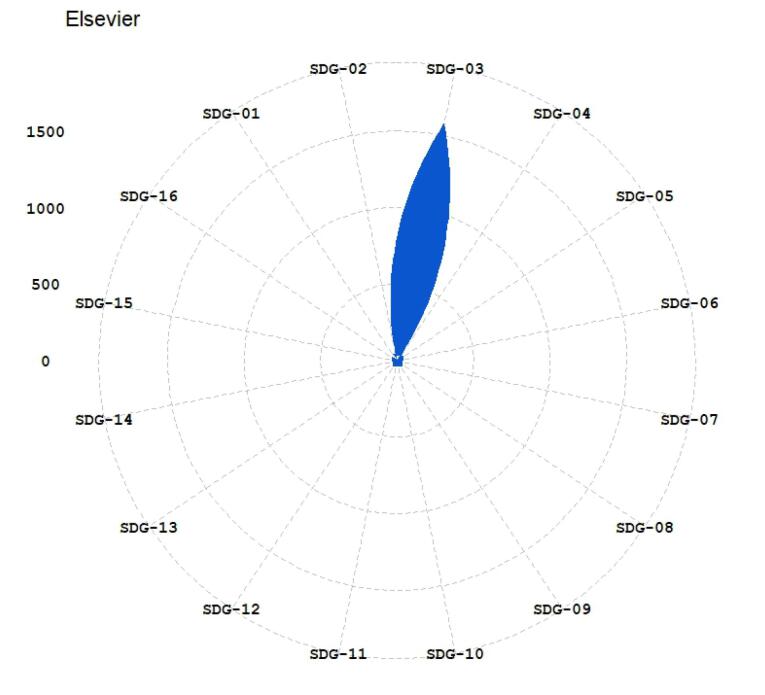
The published papers in hijacked journals and their map to SDGs (using Elsevier)

**Figure 3:**
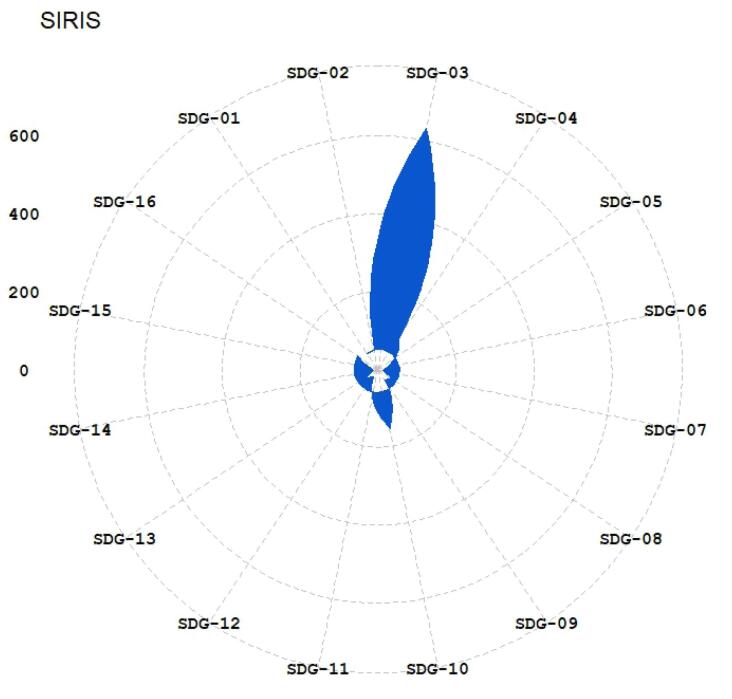
The published papers in hijacked journals and their map to SDGs (using SIRIS)

 The published papers in the hijacked version of medicine journals have been mapped to SDGs using the three methods (Aurora, Elsevier, and SIRIS). The results have been illustrated in Figures 1-3. The 17 sustainable goals are represented using these acronyms in the figures: SDG 1 (No poverty in any form), SDG 2 (Food security and sustainable agriculture), SDG 3 (Well-being for all ages), SDG 4 (Quality of education and lifetime learning), SDG 5 (Gender equality), SDG 6 (Access to water), SDG 7 (Access to sustainable energy), SDG 8 (Sustainable economic growth), SDG 9 (Sustainable infrastructure, industrialization, and innovation), SDG 10 (Reduction of inequality), SDG 11 (Sustainable cities), SDG 12 (Sustainable production), SDG 13 (Combating climate change), SDG 14 (Sustainably consideration for oceans, seas, and life below water), SDG 15 (Sustainable forests and land), SDG 16 (Peaceful societies), and SDG 17 (Global partnership to reach sustainable development). The SDGs’ descriptions are based on Gigliotti and colleagues’ research.^31^

Table 2 provides the top detected SDGs based on each method. At least two methods identified SDG 01, SDG 03, SDG 11, and SDG 16 as the top-mentioned SDGs in the published papers. SDG 02, SDG 04, SDG 05, SDG 10 and SDG 13 have been identified as top mentioned by one method. This suggests that the hijacked version of medicine journals impacts sustainable development-related research by publishing studies that fit in “good health and well-being”, no poverty, “sustainable cities and communities”, and “peace, justice and strong institutions” based on good evidence from at least two methods of SDG detection. Also, they influence the SDGs’ research by publishing research that fits with in zero hunger, quality education, gender equality, reduced inequality, and climate action based on evidence from an SDG detection method. This means that the hijacked versions of medicine journals could hurt the scientific ranks of research centers and universities, especially on SDG 01, SDG 03, SDG 11, and SDG 16. There are also risks of propagation of errors and pseudoscience related to the mentioned SDGs because hijacked journals do not conduct peer review. Lack of peer review could hurt the progress of SDGs and pose risks, especially in health-related goals.

**Table 2:** The top identified SDGs in each method

**Aurora**	**Elsevier**	**SIRIS**
SDG-03	SDG-03	SDG-03
SDG-01	SDG-01	SDG-10
SDG-13	SDG-05	SDG-04
SDG-11	SDG-11	SDG-11
SDG-02	SDG-16	SDG-16

**Figure 4:**
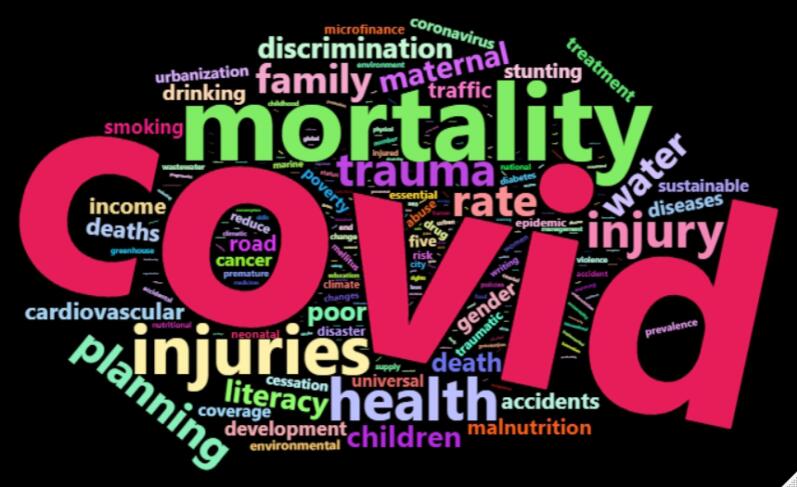
The word cloud of SDGs terms in the papers (using Aurora)

**Figure 5:**
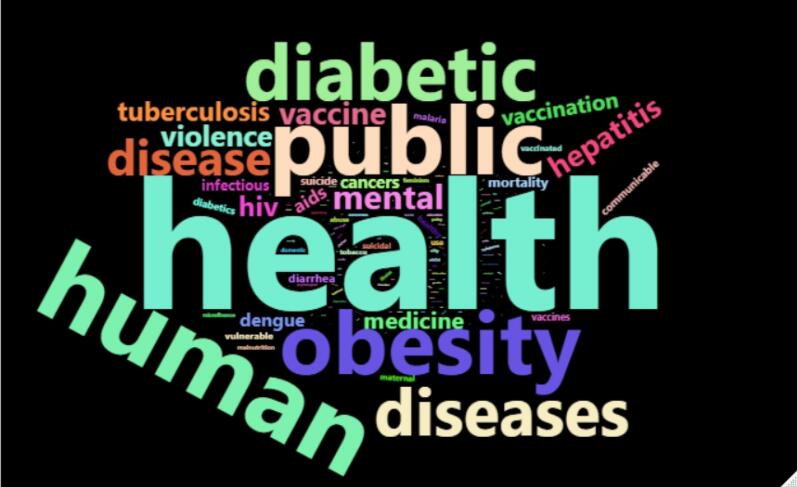
The word cloud of SDGs terms in the papers (using Elsevier)

**Figure 6:**
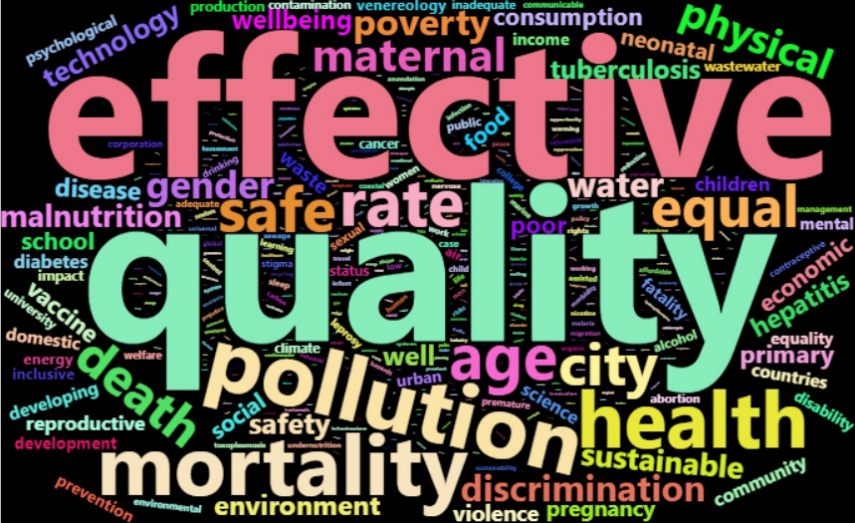
The word cloud of SDGs terms in the papers (using SIRIS)

Figures 4-6 illustrate the papers’ most frequently used SDG terms by considering each method. These keywords indicate how published papers in the hijacked journals are related to SDG science. The papers discuss COVID-19, diabetes, obesity, hepatitis, vaccine, healthcare quality, etc.

## Discussion

 The findings indicate that the hijacked version of the medicine journal publishes manuscripts that fit SDGs categories and wastes time and effort from research centers and universities for publishing papers in non-authenticated journals. This highlights the harm hijacked journals can impose on science, researchers, universities, and countries. The published papers in the hijacked journals will not impact science positively and may affect the research integrity reported as these journals do not have peer review and are managed by cybercriminals.

 The analysis (Figure 1-6) indicates that hijacked versions of medicine journals impact SDG-related research, especially on SDG 01, SDG 03, SDG 11, and SDG 16. The analysis indicates the top supported SDGs in the hijacked version of medicine journals is SDG-03 (Ensure healthy lives and promote well-being for all at all ages). This confirms that hijacked versions of medicine journals usually respect the main original journals’ aim and scope. Dadkhah et al.’s analysis also corroborated the finding that hijacked versions of medical journals often align with the original journals’ purpose and scope.^23^ However, some of the published papers may be out of the scope of the original journals, as hijackers try to publish papers in any field in the hijacked version to increase their income. As these papers will not be indexed in bibliographic databases, the effort of researchers will be wasted, and parts of sustainable development-related research will be lost. In sequence, the SDGs ranking of related institutions will be negatively impacted. While the OA model supports the progress of SDGs by providing access to research findings, hijacked journals create a barrier for SDGs because hijacked journals are not indexed in bibliographic databases such as WoS and Scopus.

 Most of the victims of hijacked journals come from India, Indonesia, Nigeria, Ethiopia, the Philippines, Thailand, and Iraq.^23^ Sustainable development is key for all of these countries, and hijacked journals can harm these countries critically. These countries also have limited GDP per capita, and the funds going to hijacked journals can negatively impact their sustainable development.

 Some of the victim universities are credible and ranked. Some have good rankings in the QS, nirf.org, etc.,^23^ suggesting that the problem of hijacked journals is not limited to low-ranking universities. Credible universities shape most of the victims. This shows the complexity of detecting hijacked journals and highlights the need for a national and international plan to deal with this problem.

## Conclusion

 The current study explored the potential repercussions of hijacked journals on sustainable development research. The results indicate that hijacked journals can waste the research budgets of universities and research centers and might disseminate research with errors. Hijacked journals negatively impact the development of science in an area. It is important that research centers and universities have to plan for the education of researchers and develop related tools to detect hijacked journals. The research published in hijacked medicine journals may be key for sustainable development, so it is important to have policies and guidelines to prevent the establishment of hijacked journals.

## Appendix

 For this research, the R language^32^ and its packages have been used.^33-43^

## AI Usage

 The AI tools have been used to improve readability in some parts of paper. The other usage of AI tools has been declared in methodology section.

## Limitations and future research

 The current research only inspected the hijacked version of medicine journals as the case study, and the results are limited to them. Future research should analyze hijacked journals in other fields. The results of this study are based on analysis of textual data, and it may contain tolerance. In data science analysis, tolerance is usual, especially when data are unstructured and dirty.

## Competing Interests

 None declared.

## Ethical Approval

 Not applicable.
